# Ectopic Liver Tissue Formation in Rats with Induced Liver Fibrosis

**DOI:** 10.5195/cajgh.2014.180

**Published:** 2014-12-12

**Authors:** Bauyrzhan Umbayev, Andrey Tsoy, Tamara Shalakhmetova, Sholpan Askarova

**Affiliations:** 1 Center for Life Sciences, Nazarbayev University, Astana, Kazakhstan; 2 Department of Biology and Biotechnology, Al-Farabi Kazakh National University, Almaty, Kazakhstan

**Keywords:** ectopic liver tissue, chronic liver fibrosis, transplantation

## Abstract

**Introduction:**

The possible alternative approach to whole-organ transplantation is a cell-based therapy, which can also be used as a “bridge” to liver transplantation. However, morphological and functional changes in the liver of patients suffering from chronic liver fibrosis and cirrhosis restrict the effectiveness of direct cell transplantation. Therefore, extra hepatic sites for cell transplantation, including the spleen, pancreas, peritoneal cavity, and subrenal capsule, could be a useful therapeutic approach for compensation of liver functions. However, a method of transplantation of hepatocytes into ectopic sites is needed to improve hepatocyte engraftment. Previously published data has demonstrated that mouse lymph nodes can support the engraftment and proliferation of hepatocytes as ES and rescue Fah mice from lethal liver failure. Thus, the aim of the study was to evaluate the engraftment of i.p. injected allogeneic hepatocytes into extra hepatic sites in albino rats with chemically induced liver fibrosis (LF).

**Materials and methods:**

Albino rats were randomly divided into 4 groups: (1) intact group (n = 18); (2) rats with induced LF (n = 18); (3) rats with induced LF and transplanted with hepatocytes (n = 18); (4) as a control, rats were treated with cyclosporine A only (n = 18). In order to prevent an immune response, groups 2 and 3 were subjected to immunosuppression by cyclosporine A (25 mg/kg per day). LF was induced using N-nitrosodimethylamine (NDMA), i.p., 10 mg/kg, three times a week for 4 weeks and confirmed by histological analysis of the liver samples. Hepatocytes transplantation (HT) was performed two days after NDMA exposure cessation by i.p. injection of 5×106 freshly isolated allogeneic hepatocytes. Liver function was assessed by quantifying blood biochemical parameters (ALT, AST, GGT, total protein, bilirubin, and albumin) at 1 week, 1 month, and 2 months after hepatocytes transplantation (HT). To confirm a hepatocytes’ engraftment, we conducted immunohistochemical staining against HepPar1.

**Results:**

We observed a 30% mortality rate among rats with LF within 1 week after NDMA exposure cessation, while 100% of animals with HT survived. ALT, AST, and GGT activities and bilirubin levels were markedly elevated in blood samples of LF rats compared to the control animals. However, HT significantly improved ALT, AST, and GGT activity as well as bilirubin levels. We also observed decreased levels of total protein and albumin in the blood serum of rats with LF, while HT normalized these parameters. At the same time, we have not detected any statistical differences of the studied parameters in the group 4, which was treated with Cyclosporine A only, compared with the intact animals. HepPar1 immunohistochemical staining of the different tissue sections demonstrated the presence of engrafted hepatocytes, mainly within enlarged Peyer’s patches (aggregated lymphoid nodules in the lowest portion of the small intestine).

**Conclusion:**

The results of our study provide evidence that HT improves animal survival and liver functions. One potential reason for these results is that ectopic hepatic mass inside the Peyer’s patches can rescue rats from liver failure.

